# The aftermath of adverse events in Spanish primary care and hospital health professionals

**DOI:** 10.1186/s12913-015-0790-7

**Published:** 2015-04-09

**Authors:** José Joaquín Mira, Irene Carrillo, Susana Lorenzo, Lena Ferrús, Carmen Silvestre, Pastora Pérez-Pérez, Guadalupe Olivera, Fuencisla Iglesias, Elena Zavala, José Ángel Maderuelo-Fernández, Julián Vitaller, Roberto Nuño-Solinís, Pilar Astier, on behalf of the Research Group on Second and Third Victims

**Affiliations:** Departamento de Salud Alicante-Sant Joan, Alicante, Spain; Universidad Miguel Hernández, Elche, Spain; Hospital Universitario Fundación Alcorcón, Madrid, Spain; Consorci Sanitari Integral, L’Hospitalet de Llobregat, Barcelona, Spain; Atención Primaria Comarca Donostia, Donostia, Spain; Observatorio para la Calidad del Sistema Sanitario en Andalucía, Sevilla, Spain; Servicio Madrileño de Salud, Madrid, Spain; Castilla La Mancha Health Service (SESCAM), Toledo, Spain; Hospital Universitario Donostia, Donostia, Spain; Gerencia de Atención Primaria de Salamanca, Castilla y León Health Service (SACYL), Salamanca, Spain; Inspección Médica, Elche, Spain; Basque Institute for Healthcare Innovation (O + Berri), Bilbao, Spain; Medicina de Familia y Comunitaria, Centro de Salud Caspe, Sector Alcañiz, Aragon Health Service (SALUD), Zaragoza, Spain

**Keywords:** Patient safety, Adverse events, Medical errors, Catastrophic loss, Healthcare providers, Primary care, Hospital

## Abstract

**Background:**

Adverse events (AEs) cause harm in patients and disturbance for the professionals involved in the event (second victims). This study assessed the impact of AEs in primary care (PC) and hospitals in Spain on second victims.

**Methods:**

A cross-sectional study was conducted. We carried out a survey based on a random sample of doctors and nurses from PC and hospital settings in Spain. A total of 1087 health professionals responded, 610 from PC and 477 from hospitals.

**Results:**

A total of 430 health professionals (39.6%) had informed a patient of an error. Reporting to patients was carried out by those with the strongest safety culture (Odds Ratio –OR- 1.1, 95% Confidence Interval –CI- 1.0-1.2), nurses (OR 1.9, 95% CI 1.5-2.3), those under 50 years of age (OR 0.7, 95% CI 0.6-0.9) and primary care staff (OR 0.6, 95% CI 0.5-0.9). A total of 381 (62.5%, 95% CI 59-66%) and 346 (72.5%, IC95% 69-77%) primary care and hospital health professionals, respectively, reported having gone through the second-victim experience, either directly or through a colleague, in the previous 5 years. The emotional responses were: feelings of guilt (521, 58.8%), anxiety (426, 49.6%), re-living the event (360, 42.2%), tiredness (341, 39.4%), insomnia (317, 38.0%) and persistent feelings of insecurity (284, 32.8%). In doctors, the most common responses were: feelings of guilt (OR 0.7 IC95% 0.6-0.8), re-living the event (OR 0.7, IC95% o.6-0.8), and anxiety (OR 0.8, IC95% 0.6-0.9), while nurses showed greater solidarity in terms of supporting the second victim, in both PC (p = 0.019) and hospital (p = 0.019) settings.

**Conclusions:**

Adverse events cause guilt, anxiety, and loss of confidence in health professionals. Most are involved in such events as second victims at least once in their careers. They rarely receive any training or education on coping strategies for this phenomenon.

**Electronic supplementary material:**

The online version of this article (doi:10.1186/s12913-015-0790-7) contains supplementary material, which is available to authorized users.

## Background

Adverse events (AEs) that cause lesions, or other types of harm or suffering in patients are also the cause of disturbance in the work, family and personal life of health professionals involved in the event [1]. These professionals are referred to as the second victims of these AEs. Second victims have been defined by Susan Scott et al. [2] as healthcare team members involved in an unanticipated patient event, in a medical error and/or a patient-related injury who become victimized in the sense that the team member is traumatized by the event.

In 1984, David Hilfiker [3] had the courage to describe his personal feelings as a second victim after a clinical error. Some years later, Frederick van Pelt [4] also described his personal experience and how he was given support to cope with the situation. Other authors have also described the experience of professionals as second victims [5,6].

David L. B. Schwappach and Till A. Boluarte [7] and Andrew White et al. [8] in 2008, Reema Sirriyeh et al. [9] in 2010, Barbara Brandom et al. [10] in 2011, Deborah Seys et al. [11] in 2012 and Elwahab and Doherty [12] in 2014 have reviewed studies published on second victims. With some exceptions [13,14], research in this field has been carried out using questionnaires or interviews with small samples of professionals. From this research, it seems that, when there is an AE, health professionals change their way of interacting with patients, respond emotionally, become insecure and doubt their professional judgement; all this, in turn, affecting the quality of care they provide to other patients. On the other hand, the professional consequences vary depending on the duration and intensity of the aftermath.

It is recognised that strategies to cope with stress and post-traumatic-stress disorder should be a key part of second-victim care [2,8]. Further, it is suspected that there is a gender difference in the response, women being more vulnerable [8]. Most research to date, however, has been conducted in North America. Moreover, there have been few studies on the aftermath of AEs in primary care (PC).

In Spain, there have hardly been any studies on this topic, and there is little experience of providing support to second victims. The Calitè research group [15], using qualitative consensus group techniques, has produced 10 recommendations for hospital managers to raise their awareness of the effects of AEs on healthcare staff. Further, some hospitals in Catalonia have started to design proposals for interventions to tackle this problem.

The ENEAS and APEAS studies on adverse effects in hospitals [16] and PC [17] in Spain found that AEs affect almost 10% of hospitalised patients per year (1 in 10 of these events having permanent or fatal consequences), while 6.7% of PC patients experience more than 1 AE per year. However, in Spain, the scale of the problem in terms of second victims is unknown, as is the impact on these health professionals. Accordingly, the objective of this study was to assess the effect of AEs that occur in PC and hospital settings in Spain on health professionals (second victims) in personal and professional terms.

## Methods

A cross-sectional study was conducted. The target population was composed of doctors and nurses of hospitals and PC health centres in the health services of Andalusia, Aragón, Castilla La Mancha, Castilla y León, Cataluña, Valencia, Madrid and the Basque Country, that is, 8 out of the 17 Spanish autonomous regions. These eight regional health services provide health cover to 76% of the Spanish population and the corresponding regions generated 78% of the GDP in 2013 [18]. The hospitals that participated in the study handled 75% of all hospital admissions and the health districts 75% of all PC consultations in 2012, according to the latest data of the Spanish Ministry of Health [19,20].

We carried out an online survey, inviting a randomly-selected sample of health professionals from these eight regions to participate. Multistage sampling was used, stratifying by level of care (PC and hospital) and autonomous region. For a level of significance of 95% and maximum estimation error of 3% with p = q = 0.50 (20% refusing replay), the goal was to interview a minimum of 1340 healthcare professionals, 670 working in hospitals and 670 in PC settings, from across the regions in proportion to their population. The sample size was increased to 2677 interviewees, given that we expected a response rate of around 50%. Together with the invitation to participate, candidate health professionals were provided with information regarding the objectives of the research and instructions on completion of the forms. We underlined that their participation was important and that their responses would be kept confidential, but also that it was voluntary and requested their informed consent. The fieldwork was carried out between May and July 2014, and the same procedure was followed in the eight participating health services. The study was approved by the Clinical Research Ethics Committees of the PC centres of Valencia (CREC APCV) and the Alcorcón Foundation University Hospital (CREC HUFA).

An AE was defined in line with the World Health Organization’s definition [21], as an incident that results in harm to a patient that is an unexpected and unintentional clinical result of healthcare, but which may or may not be related to a clinical error. Further, for a serious AE, we used the classification proposed by Field et al. [22].

### Questionnaire

The questionnaire used was developed by consensus among the research team considering published reviews [7-11], the study of the Calité group in Spain [15] and questions used in studies in North America [2,8,13] and Norway [14]. The questionnaire was structured into three sections assessing the following variables: the effect of safety culture, as a modulator of the impact of AEs on professionals (6 items for PC and 7 items for hospitals); the experience of professionals in informing patients about AEs and the frequency and intensity of the most common personal- and work-related problems among second victims and the type of support received. The safety culture items were chosen from validated questionnaires used in previous Spanish studies on Safety Culture [23,24]. These were identical to other questionnaires used in studies conducted in other countries [25]. The questions regarding the experience of second victims were posed in such a way that they did not pry into the personal experience of the interviewee, asking about their awareness of the personal and professional problems of second victims, and whether they had experience of these problems, either directly or through a close colleague (without specifying which). A score for the short scale on safety culture (5 items) was calculated by summing the scores on all the items (maximum of 25 points). The questionnaire is included as Additional file 1.

### Data analysis

Relationships between qualitative variables were assessed using the chi-square test, while Student’s t-tests were used to explore responses to questions as a function of: age (< or ≥ 50 years), sex, professional group (doctor or nurse), and whether they had received previous training in informing patients about AEs. This analysis was conducted separately for the PC and hospital staff. Logistic regression and the ENTER method were employed to assess the predictive capacity of the variables age, level of care, professional group, safety culture and sex for, first, the likelihood patients being informed of an AE, and second, the intensity attributed to the emotional problems of second victims. Differences were considered statistically significant when p < 0.05. The SPSS version 20.0 (IBM SPSS, Inc, Chicago, IL, USA) was used for this analysis.

## Results

A total of 1087 health professionals completed the questionnaires, 610 and 477 working in PC and hospitals, respectively. Women (N = 777) made up 72% of the sample as expected due to the gender relation in healthcare professions. Almost all those who completed the questionnaires had, at least, 3 years of professional experience (Table 1). Fifty-three doctors (16%) and 48 nurses (18.1%) in PC, and 39 doctors (18.7%) and 45 (19.6%) nurses in hospitals had received training on how to inform patients about AEs before the study. A total of 938 (86.3%) of participants reported having witnessed a patient safety incident within the previous 5 years. Just over half, 628 (57.8%), had reported a serious AE (Table 2).

**Table 1:** **Description of the sample**

	**PRIMARY CARE (N = 610)**		**HOSPITAL (N = 477)**	
	**N**	**%**	**N**	**%**
**Professional group**				
Doctors	332	54.4	209	43.8
Nurses	265	43.4	230	48.2
Other	13	2.1	38	7.9
**Age** (years)				
<30	10	1.6	21	4.4
31-50	310	50.8	280	58.7
51-70	287	47.0	172	36.1
DK/NR	3	0.5	4	0.8
**Sex**				
Male	151	24.8	145	30.4
Female	451	73.9	326	68.3
DK/NR	8	1.3	6	1.3
**Professional experience** (years)				
<1 - 3	3	0.5	14	2.9
>3	603	98.9	460	96.4
DK/NR	4	0.7	3	0.6
**Frequency of patient safety incidents**				
Reports of safety incidents (near misses)	465	76.2	439	92.0
Reports of serious adverse events for one or more patients	287	47.0	341	75.4
**Service (department/unit, etc.)**				
Medical	--		185	38.8
Surgical	--		92	19.3
Central	--		82	17.2
Other	--		105	22.0
DK/NR	--		13	2.7
DK/NR: Do not know or no response

**Table 2:** **Personal experience of patient safety**

	**Primary care (N=597)**					**Hospitals (N=439)**				
**In the previous 5 years**	**Doctors**	**%**	**Nurses**	**%**	**P=**	**Doctors**	**%**	**Nurses**	**%**	**P=**
In my hospital, I know of cases that could be considered near misses (incidents that could have led to serious adverse events but which were corrected in time).	251	75.6	207	78.1	0.533	193	92.3	216	93.9	0.645
I know of cases of adverse events with serious consequences for one of more patients.	174	52.4	105	39.6	0.002	168	80.4	148	64.3	0.001
I have been personally involved in informing patients who have suffered an adverse event (or their relatives)	155	46.7	66	24.9	0.001	133	63.6	66	28.7	0.001
I know of cases of professionals who have suffered emotionally after an AE in a patient.	223	67.2	158	54.9	0.008	162	77.5	184	68.7	0.140
I know of cases of health professionals who have had work-related problems due to an AE.	99	29.8	72	27.2	0.535	64	30.6	64	27.8	0.590
In your experience, what happens when a patient who has suffered an adverse event is informed?*										
The patients accepts the explanation given	239	84.8	179	87.3	0.503	168	91.8	164	84.1	0.033
The relationship with the patient worsens	77	27.5	50	25	0,612	36	19.8	63	32.8	0.006
The patient files a formal complaint	28	10.1	34	16.8	0.043	45	25.1	42	22.6	0.652
The patient responds aggressively	39	14.1	36	17.7	0.345	21	11.9	37	19.9	0.052

*Only doctors and nurses who had the experience. They are not mutually exclusive answers.

Frequency of patient safety incidents and impact on second victims.

### Patient safety culture

The mean score on the safety culture subscale was 16.9 (SD 3.6, 95% CI 16.6-17.1) (Additional file 2: online Table 1). Higher scores were obtained by hospital staff (17.5, SD 3.4 versus 16.4, SD 3.6, by those in PC; p = 0.001) and by nurses (17.2, SD 3.6 versus 16.6, SD 3.5, by doctors; p = 0.005).

The probability of there being any serious AEs for one or more patients in the following 12 months was estimated to be low or very low by 229 (38%) and 303 (65.4%) care providers in PC and hospitals respectively. However, the percentage rating this risk as low or very low decreased with increasing scores on the safety culture scale, among both PC (p = 0.001) and hospital (p = 0.004) staff. In the case of hospitals, 227 health professionals (49.1%) considered the probability of an AE in the next 12 months in their own department/unit to be low or very low, the perceptions of medical and surgical staff being similar (91, 50.2% and 45, 48.9%, respectively).

### Open disclosure experience

Almost half of the doctors in PC and 64% of doctors working in hospitals reported having informed a patient of an AE (Table 2). The probability of having this experience was higher among those with the strongest safety culture (OR 1.1, 95% CI 1.0-1.2), nurses (OR 1.9, 95% CI 1.5-2.3), women (0.6, 95% CI 0.5-0.8), those aged under 50 years old (OR 0.7, 95% CI 0.6-0.9) and those working in PC (OR 0.6, 95% CI 0.5-0.9). Approximately one third of both PC and hospital staff (228, 37.3%, and 158, 33.1%, respectively) believed that patients in their centre were told when there was an AE, doctors and nurses from PC (p = 0.070) and hospitals (p = 0.823) holding the same opinion.

All respondents, both from PC and hospitals, reported that patients told about AEs generally responded well (Table 2). However, 240 (39.4%) and 179 (37.6%) of those from PC and hospitals, respectively, indicated that patients might respond very badly to being told about an AE and that this could affect their future relationship with health professionals caring for them. We also observed that the higher the score on the safety culture scale, the more the professional favoured the idea of keeping patients properly informed about AEs (p = 0.032).

A total of 555 (91%) and 429 (89.9%) health professionals in PC and hospitals, respectively, said that they would be interested in receiving specific training on how to tell patients about AEs. This type of training was of similar interest to both professional groups in PC (p = 0.537), but of more interest to nurses (218, 95.6%) than doctors in hospitals (182, 89.7%) (p = 0.028).

### Second victim experience

A total of 381 (62.5%, 95% CI 59-66%) health professionals in PC and 346 (72.5%, 95% CI 69-77%) in hospitals reported having suffered the second-victim experience (themselves or through colleagues) in the previous 5 years (Table 2). In PC, doctors reported knowing more people affected than did nurses (223, 67.2% vs 149, 56.2%, p = 0.008), while in hospitals the professional groups reported similar rates of knowing cases of second victims (162, 77.5% versus 163, 70.9%, p = 0.140). The professionals from medical, surgical and central services reported similar rates of second victims in their respective areas (p = 0.488).

The perception of the support received by second victims was similar in PC and hospitals. In hospitals, 66 (13.4%) professionals indicated that second victims received psychological counselling and 222 (46.5%) that second victims received support from their own department/unit. Both professional groups from both levels of care reported similar rates of psychological counselling for second victims, but a higher proportion of nurses than doctors reported support for second victims from their own colleagues, both in PC (p = 0.019) and hospitals (p = 0.019).

Almost a third indicated that second victims were unable to continue working after an AE and required time off (Table 3). In PC, the reported work-related consequences for second victims were similar in the two professional groups, while in hospitals, nurses reported a higher rate of second victims taking time off work and being transferred to different departments/units. Staff who considered that the probability of an AE was low or very low reported the fewest cases of second victim events (p = 0.001).

**Table 3:** **Professional consequences observed in second victims**

	**Primary care (N=458)**					**Hospital (N=360)**				
	**Doctors (N=267)**		**Nurses (N=191)**			**Doctors (N=182)**		**Nurses (N=178)**		
	**N**	**%**	**N**	**%**	**P=**	**N**	**%**	**N**	**%**	**P=**
Required time off work	74	27.8	52	27.7	1.00	25	13.7	43	24.2	0.017
Requested transfer to a different department/unit or health centre	41	15.4	36	18.8	0.288	18	9.9	45	25.1	0.001
Left the profession	8	3.0	5	2.6	1.00	3	1.7	3	1.7	1.00

The most common emotional responses were: feelings of guilt (521, 58.8%), anxiety (426, 49.6%), re-living the event, again and again (360, 42.2%), tiredness (341, 39.4%), insomnia (317, 38.0%), persistent doubts about what to do in each case and whether clinical decisions are correct (284, 32.8%) and feeling dazed, confusion and difficulties concentrating in work (260, 29.9%) (Table 4). These emotional responses were more intense among doctors females than males (guilt *t*-test 3.0, p = 0.003; anxiety *t*-test 2.5, p = 0.01; re-living the event, again and again *t*-test 3.3, p = 0.001; tiredness *t*-test 2.8, p = 0.006; insomnia *t*-test 2.7, p = 0.007; persistent doubts about what to do in each case and whether clinical decisions are correct *t*-test 4.3, p < 0.001; and feeling dazed, confusion and difficulties concentrating in work *t*-test 3.1, p = 0.002). Doctors reported a greater intensity of these emotional disturbances in second victims (feelings of guilt, OR 0.7, 95% CI 0.6-0.8; re-living the event, OR 0.7, 95% CI 0.6-0.8; and anxiety, OR 0.8, 95% CI 0.6-0.9). The most common professional consequences for second victims were: concerns about the legal consequences of an AE, the potential damage to their professional standing due to the incident, having to say sorry to patients and having to inform to managers (Table 5). In PC, the legal consequences of an AE (7.5, SD 2.2 vs 7.8, SD 1.9, p = 0.046) and damage to their professional standing (6.7, SD 2.3 vs 7.3, SD 2.2, p = 0.030) were of more concern to those under 50 years of age, while in hospitals, this younger age group were more worried about their standing (6.5, SD 2.6 versus 7.2, SD 2.3, p = 0.002).

**Table 4:** **Emotional response commonly observed in second victims**

	**Primary care**					**Hospital**				
	**Doctors (N = 332)**		**Nurses (N = 265)**			**Doctors (N = 209)**		**Nurses (N = 230)**		
**In the case that you or a colleague has been involved in a serious adverse event, indicate the frequency of the following responses**	**Mean**	**SD**	**Mean**	**SD**	**P=**	**Mean**	**SD**	**Mean**	**SD**	**P=**
Feeling dazed/confusion/difficulty concentrating on work, in the days after an adverse event	2.3	0.8	2.0	0.8	0.000	2.0	0.7	2.1	0.9	0.086
Feelings of guilt	2.9	0.7	2.5	0.8	0.000	2.7	0.8	2.9	0.9	0.003
Pessimism about life/sadness	2.2	0.8	2.0	0.8	0.001	2.1	0.7	2.2	0.9	0.382
Tiredness	2.5	0.8	2.2	0.8	0.000	2.3	0.8	2.3	0.9	0.709
Anxiety	2.7	0.8	2.4	0.9	0.001	2.4	0.8	2.6	0.9	0.075
Insomnia/trouble sleeping well	2.5	0.8	2.2	0.8	0.005	2.2	0.8	2.4	0.9	0.084
Re-living the event, again and again	2.6	0.8	2.2	0.8	0.000	2.3	0.8	2.5	0.9	0.187
Anger and mood swings at work	2.1	0.7	1.9	0.7	0.029	2.0	0.7	2.0	0.8	0.847
Anger and mood swings at home	2.2	0.7	2.0	0.8	0.005	2.1	0.7	2.1	0.8	0.772
Constant doubts about what to do and whether clinical decisions are correct	2.4	0.7	2.1	0.7	0.000	2.2	0.7	2.3	0.8	0.165
Concern about loss of standing among colleagues	1.6	0.6	1.7	0.7	0.806	1.6	0.7	1.8	0.8	0.008
Concern about loss of standing among patients	1.9	0.6	1.7	0.7	0.020	1.5	0.6	1.7	0.8	0.003
Questioning whether to leave the profession	1.8	0.8	1.5	0.7	0.000	1.5	0.6	1.6	0.8	0.456
Response options for all items from 1 (never) to 4 (always)

**Table 5:** **Relational difficulties commonly observed in second victims**

	**Primary care**					**Hospital**				
	**Doctors (N = 332)**		**Nurses (N = 265)**			**Doctors (N = 209)**		**Nurses (N = 230)**		
**In event of a clinical error, indicate how often those involved are likely to do the following**	**Mean**	**SD**	**Mean**	**SD**	**P=**	**Mean**	**SD**	**Mean**	**SD**	**P=**
Say sorry to patients (or their relatives)	6.9	2.3	7.1	2.5	0.437	6.1	2.9	6.0	2.9	0.605
Face legal action	8.0	1.9	7.3	2.2	0.000	7.8	2.0	7.7	2.2	0.734
Lose professional standing	7.2	2.2	7.0	2.4	0.422	6.6	2.4	7.3	2.4	0.006
Inform the clinical manager of the health centre/hospital of the error	5.4	2.8	5.5	2.6	0.711	5.5	2.8	5.1	2.7	0.223
Come into conflict with colleagues (reproach or criticism)	4.6	2.7	4.6	2.5	0.778	5.0	2.6	5.3	2.8	0.299
Frequency scale from 0 (never) to 10 (always)										

The great majority, 583 (92.3%) respondents from PC and 430 (90.1%) from hospitals, indicated that they would be interested in receiving specific training to cope better with the impact of AEs among professionals. In PC, the two professional groups indicated similar rates of interest in this type of training (p = 0.885), while in hospitals there was more interest among nurses (223, 98.2%) than doctors (176, 87.6%) (p = 0.001). Both age groups (p = 0.165) and both professional groups (p = 0.757) were similarly uncomfortable about saying sorry to a patient for a clinical error.

## Discussion

Ideally, health professionals would never be involved in safety incidents that result in an AE with serious consequences for one or more patients. However, clinical practice is not risk free. The results of this study highlight that 8 out of 10 health professionals working in PC and nearly all those in hospitals in Spain have witnessed a patient safety incident at some point in their career. This study also indicates that 6 out 10 health professionals in Spain have experience of the emotional and professional difficulties of professionals (themselves or close colleagues) in the aftermath of an AE in a patient. As expected [13], the emotional responses of females in these cases were more negative. That is, our data suggest that most health professionals have directly or indirectly been involved in this type of event. Therefore, it seems reasonable that they should be prepared for such an eventuality [26]. To look the other way, ignoring this reality, is not an adequate response [1].

In this case, three quarters of doctors and nurses had an experience as second victims. This figure is similar to those found in other studies in Europe [27,28], Canada [29] and USA [13]. This result confirms the magnitude of the second victim phenomenon for all healthcare organizations.

Most studies on the impact of AEs on health professionals (second victims) and on how to cope with this situation have been carried out in the USA and Canada [13,29]. In Europe, fewer empirical studies have been conducted and many have focused on nurses [30-32]. Nevertheless, results [14,33,34] have tended to show that the following are common among second victims after an AE: feelings of guilt, anxiety, and concern about the consequences [2,7,9,11,13] and that, in line with in the findings of other studies, the role of colleagues and the management is crucial, especially in the early stages after an AE [8,35].

Our findings suggest that, as is the case in other countries [7,13,35], health professionals in Spain are not receiving training to coping with this aftermath. They do not receive the necessary support for coping with the task of telling patients about AEs. This study has explored the point of view of doctors and nurses working in two levels of care: PC and hospitals, providing information that is qualitatively different to the data available previously. Nurses seem to show greater solidarity with second victims, contributing to reducing the branding of second victims with a scarlet letter in the workplace.

The experience of second victims is related to post-traumatic stress disorder but with some extra factors: doubts regarding informing patients, colleagues and managers about what has happened, fear of the legal consequences of AEs, and concerns about a loss of standing (feeling that event will mark them forever, a scarlet letter) [13]. Almost half of the participants had experience of telling a patient about an error. However, we found that this task is most likely to be undertaken by nurses, younger health professionals and those with a strongest safety culture and that it occurs more often in PC than in hospitals. It is known that reporting an AE and explaining its causes and consequences is very difficult for any health professional [35]. In the Spanish context, doctors worry more than nurses about the negative professional consequences of AEs, and hence, it seems advisable that support programmes put into place recognise these differences. Considering the results of the Tromsø study [27], their concerns are justifiable given the fact that patients, and society as a whole, consider doctors to be directly responsible for AEs, involvement in this type of incident negatively affecting their professional standing. When interpreting these results, we should bear in mind that in Spain there are no privilege statutes to protect health professionals who disclose information regarding the circumstances of an AE, either to committees analysing the incident or to the patient who has experienced the AE [36].

Most studies and proposals to date [9,37] have focused on how to act after an AE to support second victims in hospitals. Other results suggested that there are hardly any differences between PC and hospital health staff in their responses as second victims, except in relation to the different causes and consequences of the most common AEs at each level of care [6,13]. This is important, since it can be supposed from our data that the approaches shown to be useful for providing support for second victims of AEs in the hospital setting would also be applicable to PC professionals. This is particularly relevant in countries, such Spain, with a very well developed PC system and potentially able to implement the recommendations that have arisen from other studies [38].

Considering the potential numbers of second victims in PC and hospitals, and given that to date health centre managers have hardly begun to address this issue [39], it seems advisable to take steps to raise awareness among health professionals, as well as to reinforce a safety culture. Interventions should include addressing the needs of second victims and developing crisis plans at the institutional level for when these incidents occur.

Limitations. The health professionals who did not participate in this study may not have the same safety culture and their perceptions may be different from those of the respondents. Recall biases were also possible when reporting this information. Further, we did not study the consequences for second victims in their family life and other factors that were not assessed in this study may also affect the impact of AEs on second victims. These include: the previous relationship of health professionals with the patient and their family, the number of health professionals involved in the same incident, support professionals receive from their family and friends, previous experience with this type of incident, and the media impact of the incident. Statistical differences should consider the magnitude of the differences to interpret the meaningful of these differences. Our results do not allow us to assess the prevalence of affective or post-traumatic disorders among professionals, and nor do they enable us to estimate the potential costs associated with provision of support to second victims. Lastly, while the types of interventions that might be useful to support second victims can be inferred from the results, the study was not designed with this objective.

## Conclusions

- Six out 10 health professionals in Spain have known the second victim experience.
- The experience of second victims in Spain cause guilt and anxiety but with some extra factors: doubts regarding informing patients, colleagues and managers about what has happened, fear of the legal consequences, and concerns about a loss of standing.
- In the Spanish context, doctors worry more than nurses about the negative professional consequences of AEs. There are also differences in the aftermath in PC or hospitals.
- Spanish health professionals rarely receive any training or education on coping strategies for this phenomenon

## Additional files

Additional file 1:
**Questionnaire used in the case of hospital professional.** The questionnaire used in primary care in the same changing hospital for health district. Additional file 2: Online Table 1. Responses to the safety culture subscale.

## Abbreviations

AE: Adverse event
CI: Confidence interval
CREC-APCV: Clinical Research Ethics Committees of the PC centres of Valencia
CREC-HUFA: Clinical Research Ethics Committees of the PC centres of the Alcorcón Foundation University Hospital
PC: Primary care
OR: Odds ratio
SD: Standard deviation
USA: United State of America
