# Supplementary material for: The aftermath of adverse events in Spanish primary care and hospital health professionals
Source: BMC Health Serv Res. 2015 Apr 9;15:151. doi: 10.1186/s12913-015-0790-7 (PMC4394595; doi:10.1186/s12913-015-0790-7)
Supplement: Additional file 1: — Questionnaire used in the case of hospital professional. The questionnaire used in primary care in the same changing hospital for health district. [file 12913_2015_790_MOESM1_ESM.docx]

**Additional file 1**

**Questionnaire used in the case of hospital professional. The questionnaire used in primary care in the same changing hospital for health district.**

Clinical practice is not free from risk. Research carried out in Spain show frequencies of Adverse Events (AE) of around 10% in hospitals (ENEAS Study) and 1.8% in primary care (APEAS Study). A conservative estimate would suggest that, annually, around 15% of health professionals are involved in some AE. These professionals are referred to as “secondary victims” of AEs.

In secondary victims AEs can have emotional, professional and family consequences, even in those cases in which the AE is the result of a fault in the system or the AE could not have been predicted.

In this study, funded by the *Fondo de Investigación Sanitaria* (FIS; Fund for Health Research) and by ERDF Funds, we set out to analyze the consequences of AEs for professionals as secondary victims, with a view to proposing alternatives for them to cope with such consequences.

------------------------

- RECORD: With the Record Scores button you can record your scores without sending them definitively, in case you want to continue with the questionnaire later.
- SUGGESTIONS: In case you think of options other than those offered in the questionnaire.

------------------------

Adverse Event (AE) with serious consequences is defined as: unforeseeable or unexpected event that causes in the patient (or group of patients) harm that is difficult to repair (requiring new treatment with uncertain outcome or a prolongation of their hospital stay) or impossible to repair (surgery in the wrong place, permanent disability or death) and which is a consequence of the healthcare and not of the patient’s original illness.

AEs are considered to have occurred regardless of whether the event could have been avoided or was inevitable.

This definition includes: organizational faults, wrong diagnoses and errors of prescription, of medication use or of communication between professionals or with the patient, as long as they result in harm to the patient that is difficult to repair (requiring new treatment with uncertain outcome or a prolongation of their hospital stay) or irreparable (surgery in the wrong place, permanent disability or death).

This questionnaire is made up of 51 questions grouped in nine blocks. Estimated time needed for filling it out is 15 to 20 minutes.

1. **With regard to the last 5 years, please choose the answer that best reflects your personal experience. In my hospital…**

| 1.- We have an annual patient safety training plan with two different levels: raising awareness and specific training (workshops or courses). | □ Totally agree  □ Disagree  □ Neither agree nor disagree  □ Agree  □ Totally disagree  □ Don’t know/No answer |
| --- | --- |
| 2.- We have a system for the anonymous reporting of incidents and AEs that permits the collection of information that is useful for avoiding risks to patients. |  |
| 3.- When an AE with serious consequences for a patient is detected, there always follows an analysis of its causes and how to avoid the same thing happening in the future (we learn from experience in systematic fashion). |  |
| 4.- The majority of clinical errors I have heard of are due to faults in the organization, and not to human mistakes. |  |
| 5.- The majority of AEs with serious consequences could be avoided. |  |
| 6.- Professionals who find themselves involved in an AE have Access to psychological help from the hospital for reducing the impact on them as secondary victims. |  |
| 7.- I have received training in how to inform a patient that he/she has been the victim of an AE. |  |
| 8.- When a clinical error occurs that affects a patient, he/she or his/her family is always contacted. |  |
| 9.- Informing patients about errors or faults that have no relevant effects on their treatment causes unnecessary alarm. |  |
| 10.- Informing a patient about a clinical error can cause a highly negative reaction that affects his or her subsequent relationship with the professionals attending to them. |  |
| 11.- When a serious AE occurs, the professional (or professionals) involved receive support from their own team |  |

1. **Please indicate how probable you think it is that in the next 12 months…**

| 1.- There will be an AE with serious consequences at your hospital. | □ High  □ Moderate  □ Low  □ Not at all probable |
| --- | --- |
| 2.- There will be an AE with serious consequences in your department. |  |

1. **In the case of a clinical error occurring, how likely do you think the following would happen (from 0 = Highly unlikely to 10 = Highly likely):**

| 1.- Informing the patient or his/her family about the error. | □ 1  □ 2  □ 3  □ 4  □ 5  □ 6  □ 7  □ 8  □ 9  □ 10 |
| --- | --- |
| 2.- Apologizing to the patient (or his/her family). |  |
| 3.- Being afraid of confronting the legal consequences |  |
| 4.- Being afraid of losing professional prestige. |  |
| 5.- Not knowing how to inform the person in charge of clinical matters about the error. |  |
| 6.- Having conflicts with other professionals (disapproval or criticism) |  |

1. **In the last 5 years…**

| 1.- I have seen or heard of cases at my hospital of situations that could be considered as “almost errors” (incidents that could have caused harm to a patient, but which were finally corrected in time). | □ Yes  □ No |
| --- | --- |
| 2.- I have seen or heard of cases of AEs with serious consequences for one or more patients. |  |
| 3.- I have seen or heard of cases of professionals who have been in very bad emotional states because of an AE in a patient. |  |
| 4.- I have seen or heard of cases of professionals who have had work-related problems because of an AE. |  |
| 5.- I have personally had to inform a patient (or his/her family) that he/she has been the victim of an AE. |  |

1. **From your own experience or what you have heard from others, what were the consequences of informing a patient about an avoidable AE that has affected him/her?:**

| 1.- The patient did not accept the explanations. | □ Yes  □ No |
| --- | --- |
| 2.- The relationship with the patient suffered as a result of the conversation. |  |
| 3.- The patient filed a lawsuit. |  |
| 4.- The patient reacted aggressively. |  |

1. **In the case that you or any professional you know has been involved in an avoidable AE with serious consequences, please indicate whether or not you have observed the following symptoms:**

| 1.- Bewilderment, confusion, difficulties for concentrating on one’s job in the days following the AE. | □ Never  □ Sometimes  □ Almost always  □ Always |
| --- | --- |
| 2.- Feelings of guilt. |  |
| 3.- Pessimism about life, sadness. |  |
| 4.- Tiredness. |  |
| 5.- Anxiety. |  |
| 6.- Insomnia, difficulties for getting proper sleep. |  |
| 7.- Reliving the event over and over again. |  |
| 8.- Anger and mood swings at work. |  |
| 9.- Anger and mood swings at home. |  |
| 10.- Constant doubts about what one must do in each case and whether the clinical decisions one makes are the right ones. |  |
| 11.- Loss of professional reputation among one’s co-workers. |  |
| 12.- Loss of professional reputation with patients. |  |
| 13.- Questioning oneself about whether to continue in the profession or give it up. |  |

1. **In the case that you or any professional you know has been involved in an avoidable AE with serious consequences, please indicate whether or not:**

| 1.- You/He/She has needed time off from work. | □ Yes  □ No |
| --- | --- |
| 2.- You/He/She has applied for a transfer to another department, unit or institution/health centre. |  |
| 3.- You/He/She has given up the profession. |  |

1. **Please indicate whether or not you would be interested in receiving specific training in:**

| 1.- How professionals can cope better with the consequences of AEs. | □ Yes  □ No |
| --- | --- |
| 2.- How to inform a patient that he/she has been the victim of an AE. |  |

1. **Finally, please state your:**

| 1.- AGE | □ 30 or under  □ 31 to 50  □ 51 to 70 |
| --- | --- |
| 2.- SEX | □ Man  □ Woman |
| 3.- PROFESSION | □ Doctor  □ Nurse  □ Nursing assistant  □ Porter  □ Other |
| 4.- UNIT OR DEPARTMENT | □ Medical  □ Surgical  □ X-Ray - Laboratory  □ Others |
| 5.- YEARS OF PROFESSIONAL EXPERIENCE | □ Less than 1 year  □ 1-3 years  □ Over 3 years |

If you would like to make any suggestions, please write them in this space
