# Supplementary material for: The aftermath of adverse events in Spanish primary care and hospital health professionals
Source: BMC Health Serv Res. 2015 Apr 9;15:151. doi: 10.1186/s12913-015-0790-7 (PMC4394595; doi:10.1186/s12913-015-0790-7)
Supplement: Additional file 2: Online Table 1. — Responses to the safety culture subscale. [file 12913_2015_790_MOESM2_ESM.doc]

Additional file 2

Online table 1. Responses to the safety culture subscale

|  | | Primary Care | | | |  | |  | |  | | Hospital |  | |  |  |  |  |  | |  |  |  |
| --- | --- | --- | --- | --- | --- | --- | --- | --- | --- | --- | --- | --- | --- | --- | --- | --- | --- | --- | --- | --- | --- | --- | --- |
|  | Doctors (N=332) | | | | | Nurses (N=265) | | | |  | | Doctors (N=209) | Nurses (N=230) | | |  | Primary Care (N=610) | Hospital (N=477) | | |  |  |  |
|  | Mean | | SD | | Mean | | SD | | P= | | Mean | SD | Mean | SD | | P= | Mean | SD | | Mean | SD | P= | |
| 1.1. We have an annual training plan on patient safety that works at different levels: promoting awareness and specific training (workshops and courses). | 2.9 | | 1.3 | | 3.0 | | 1.4 | | 0.641 | | 2.9 | 1.4 | 3.4 | 1.3 | | 0.000 | 2.9 | 1.4 | | 3.1 | 1.4 | 0.054 | |
| 1.2. We have an anonymous system for reporting incidents and adverse events enabling us to collect useful data to reduce risks for our patients. | 3.5 | | 1.4 | | 3.3 | | 1.5 | | 0.025 | | 3.7 | 1.4 | 4.1 | 1.2 | | 0.004 | 3.4 | 1.5 | | 3.9 | 1.3 | 0.001 | |
| 1.3. When a serious adverse event is detected, an analysis is undertaken of the causes and how to avoid such an event in the future (we systematically learn from experience). | 3.3 | | 1.3 | | 3.4 | | 1.4 | | 0.239 | | 3.5 | 1.2 | 3.8 | 1.2 | | 0.010 | 3.3 | 1.3 | | 3.6 | 1.2 | 0.001 | |
| 1.4. Most adverse events I am aware of are due to organisational failures rather than human error. | 2.8 | | 0.9 | | 2.8 | | 0.9 | | 0.759 | | 3.0 | 1.0 | 2.9 | 1.0 | | 0.096 | 2.8 | 0.9 | | 2.9 | 1.0 | 0.008 | |
| 1.5. Most serious adverse events are avoidable. | 3.8 | | 0.9 | | 3.9 | | 0.8 | | 0.017 | | 3.8 | 0.9 | 4.0 | 0.9 | | 0.103 | 3.8 | 0.9 | | 3.9 | 0.9 | 0.386 | |
| Response options from 1 (Strongly disagree) to 5 (Strongly agree) |  | |  |  | | |  | |  | |  |  |  |  | |  |  |  | |  |  |  | |
